# Broad-Spectrum Anticancer Activity and Pharmacokinetic Properties of a Prenyloxy-Substituted Indeno[1,2-*b*]indole Derivative, Discovered as CK2 Inhibitor

**DOI:** 10.3390/ph14060542

**Published:** 2021-06-05

**Authors:** Ehab El-Awaad, Robin Birus, Christelle Marminon, Zouhair Bouaziz, Laurens Ballentin, Dagmar Aichele, Marc Le Borgne, Joachim Jose

**Affiliations:** 1 Institut für Pharmazeutische und Medizinische Chemie, PharmaCampus, Westfälische Wilhelms-Universität Münster, Corrensstr. 48, 48149 Münster, Germany; ehab.elawaad@uni-muenster.de (E.E.-A.); robin.birus@uni-muenster.de (R.B.); laurens.ballentin@uni-muenster.de (L.B.); dagmar.aichele@uni-muenster.de (D.A.); 2 Department of Pharmacology, Faculty of Medicine, Assiut University, Assiut 71515, Egypt; 3 Small Molecules for Biological Targets Team, Centre de Recherche en Cancérologie de Lyon, Centre Léon Bérard, CNRS 5286, INSERM 1052, Université Claude Bernard Lyon 1, Univ Lyon, 69373 Lyon, France; christelle.marminon-davoust@univ-lyon1.fr; 4 EA 4446 Bioactive Molecules and Medicinal Chemistry, Université Claude Bernard Lyon 1, Univ Lyon, 69373 Lyon, France; zouhair.bouaziz@univ-lyon1.fr

**Keywords:** CK2 inhibitor, indeno[1,2-*b*]indole, anticancer, pharmacokinetics, LC-MS

## Abstract

Protein kinase CK2 is involved in regulating cellular processes, such as cell cycle, proliferation, migration, and apoptosis, making it an attractive anticancer target. We previously described a prenyloxy-substituted indeno[1,2-*b*]indole (5-isopropyl-4-(3-methylbut-2-enyloxy)-5,6,7,8-tetrahydroindeno[1,2-*b*]indole-9,10-dione (**4p**)) as a very potent inhibitor of CK2 holoenzyme (IC_50_ = 25 nM). Here, we report the broad-spectrum anticancer activity of **4p** and provide substantial progress on its pharmacokinetic properties. Using a cell-based CK2 activity assay and live-cell imaging of cultured A431, A549, and LNCaP cancer cell lines, cellular CK2 target engagement was shown as well as strong antiproliferative, anti-migratory and apoptosis-inducing effects of **4p**. Furthermore, evidence was found for the ability of **4p** to disrupt A549 spheroid cohesion. A series of LC-MS/MS experiments revealed high and rapid cellular uptake (intracellular concentration is approximately 5 µM after 1 h incubation) and low metabolic stability of **4p**. These results point to the value of **4p** as a potent CK2 inhibitor with promising anticancer activities and should trigger future medicinal chemistry efforts to improve the drug-like properties of this compound.

## 1. Introduction

Human protein kinase CK2, originally named “Casein Kinase II” [1], is a ubiquitously expressed serine/threonine kinase that phosphorylates several hundreds of substrates in eukaryotic cells [2,3,4]. This highly pleiotropic kinase is involved in the regulation of numerous cellular processes, including, among others, cell proliferation, cell survival, as well as gene transcription and translation [5,6,7,8,9,10]. Constitutive CK2 activity is essential to the cell, with knockouts of both the catalytically active CK2α and the regulatory CK2β subunits found lethal during embryonic development [11,12,13]. Besides its studied roles in diseases, such as viral infections [14,15], chronic inflammation [16], and neurodegenerative disorders [17], CK2 is known to be closely associated with cancer.

In cancer cells, upregulation of CK2 expression/activity allows them to proliferate strongly and circumvent apoptosis, and therefore these malignant cells become dependent on high CK2 activity for their survival. Treatment with CK2 inhibitors aims to downregulate CK2 activity in cancer cells to basal constitutive levels leading to antiproliferative and proapoptotic effects in various malignancies [18,19,20,21]. On the other hand, normal cells with constitutive CK2 activity exhibit only a modest apoptosis-inducing effect in response to downregulation of CK2 [21,22]. The continuously growing body of evidence for CK2 oncogenic potential makes this protein kinase one of the most studied targets for cancer therapy in recent decades [23,24,25]. Furthermore, the cellular level of this enzyme can serve as a prognostic biomarker in tumor diagnostics [26].

**Figure 1:**
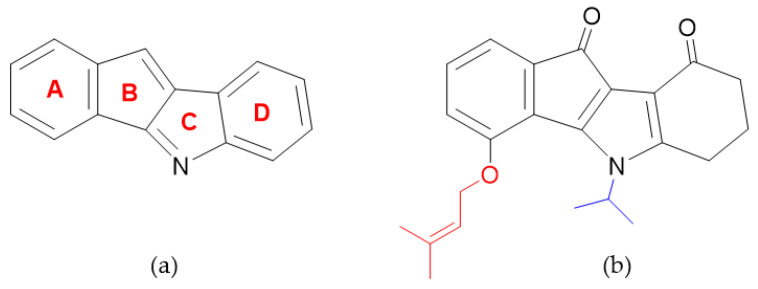
Structure of indeno[1,2-*b*]indole scaffold with the corresponding labels of the aromatic four-ring system (**a**) and **4p** (**b**) with both substitutions important for strong CK2 inhibition (i.e., prenyloxy and isopropyl groups) shown in red and blue, respectively.

The indeno[1,2-*b*]indole scaffold (Figure 1a) is known to be an interesting framework for developing pharmacologically active compounds with anticancer activities [27,28]. Several indeno[1,2-*b*]indole derivatives have been reported as promising type I inhibitors of CK2 [29,30,31,32]. The compound (5-isopropyl-4-(3-methylbut-2-enyloxy)-5,6,7,8-tetrahydroindeno[1,2-*b*]indole-9,10-dione) previously described by Gozzi et al. [33] and known as **4p** (Figure 1b), was shown to possess remarkably high in vitro inhibitory activity toward CK2 holoenzyme among indeno[1,2-*b*]indole derivatives. This interesting feature of **4p** triggered a follow-up structural study to experimentally determine the prerequisites for the observed high-affinity binding of **4p** to CK2 [34]. X-ray structures of **4p** with human CK2α or its paralog CK2α’ revealed an unusual “hydrophobic-out/oxygen-in” binding mode mainly mediated by the hydrophobic interactions through the prenyloxy group and the isopropyl substituent of the aromatic four-ring system (Figure 1b) [34]. In contrast, **4p** exhibited very weak inhibition of the breast cancer multidrug resistance ABCG2 transporter, an off-target for several protein kinase inhibitors, suggesting that this compound may have a satisfactory CK2 selectivity profile [33,34]. Furthermore, **4p** was shown in these studies to exhibit a distinct antiproliferative effect at 20 μM against the breast cancer cell line MCF-7 and to have a favorable intestinal absorption profile as derived from the high apparent permeability coefficient P_app_ in Caco-2 assay [33,34].

Although **4p** seems to be a promising CK2 targeting lead structure, there has been no thorough investigation of the compound’s anticancer activities, its cellular target engagement, or pharmacokinetic properties. In this work, we evaluated the effects of **4p** on three different cell lines, namely A431, A549, and LNCaP, which are representative models of skin, lung, and prostate cancers, respectively. These cell lines were found to be dependent on CK2 for their survival as retrieved from the cancer Dependency Map project (DepMap) database (https://depmap.org/portal, accessed on 15 April 2021). The ability of **4p** to inhibit cellular CK2 activity in these cell lines and the subsequent effects on cell proliferation, apoptosis, and migration were evaluated. Furthermore, the impact of **4p** on the integrity of 3D multicellular spheroids from the A549 cell line was monitored. We also analyzed the cellular uptake of **4p** by A431 cells and studied its metabolic stability in vitro to get valuable insight into the pharmacokinetic properties of this compound.

## 2. Results and Discussion

### 2.1. Cellular Target Engagement

**Figure 2:**
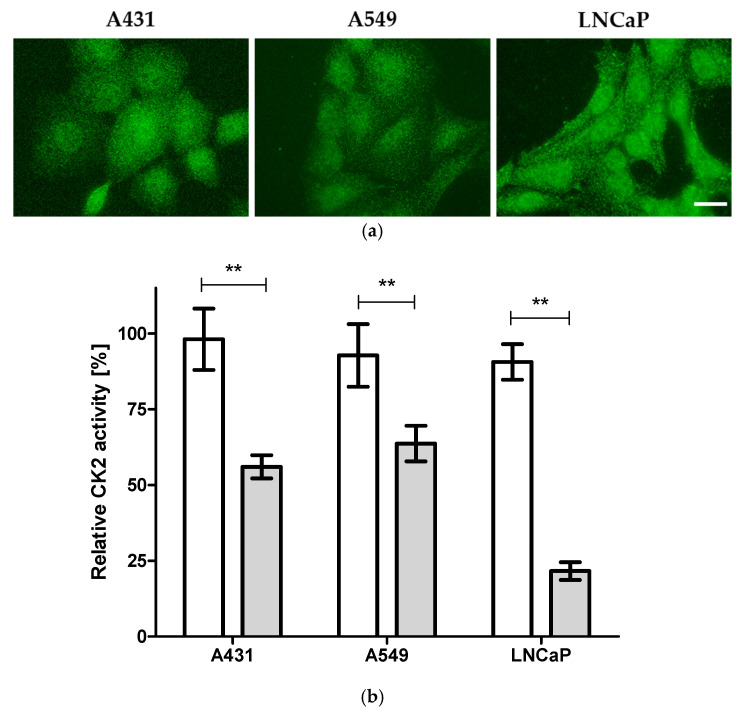
CK2 inhibition by **4p** in three different cancer cell lines. (**a**) Immunostained A431, A549, and LNCaP cells with anti-CK2α antibody (1AD9, Santa Cruz Biotechnology, Santa Cruz, CA, USA) and corresponding fluorescently labeled secondary antibody. Scale bar = 20 µM. (**b**) CK2 activity in lysates from cultured A431, A549, or LNCaP cells that were treated for 24 h with vehicle (1% DMSO) or **4p** at 1 µM (white bars) and 20 µM (grey bars) was determined in a capillary electrophoresis-based enzyme activity assay [35]. Data represent mean ± SD from three independent measurements, each performed in triplicates. ** *p* < 0.01.

First, the suitability of A431, A549, and LNCaP cell lines to study the cellular effects of CK2 inhibitors was experimentally verified by the examination of the expression of CK2α in these cell lines using immunocytochemistry. The three cell lines demonstrated strong ubiquitous CK2α expression (Figure 2a), supporting the notion that these cell lines are dependent on CK2 for their survival. Next, to determine the cellular target engagement of protein kinase CK2 by **4p**, cultured cells from A431, A549, and LNCaP cell lines were treated with **4p** at 1 and 20 µM or vehicle (1% DMSO) for 24 h followed by harvesting and lysing the cells. CK2 activity in the soluble fraction of the cell lysates was determined in a capillary electrophoresis-based CK2 activity assay as described before [35] utilizing the fluorescently labeled CK2 substrate FITC-RRRDDDSDD-NH2 (A representative electropherogram is shown in the Supplementary Materials, Figure S1). The ratio of the area under the curve (AUC) values for the peak of the substrate and the corresponding peak of the phosphorylated product was determined for each sample to calculate relative CK2 activity. Ratios for **4p** treated samples were then normalized to those of control samples. As shown in Figure 2b, lysates from cells treated with 1 µM **4p** demonstrated very weak inhibition of CK2 (<10% for all cell lines), whereas treatment with 20 µM led to statistically significant, but variable, inhibition values (44, 36, and 78% for A431, A549, and LNCaP cells, respectively). These results indicate that the determined intracellular CK2 inhibition by **4p** is, at least, partially involved in mediating the biological effect in these cell lines. Nevertheless, there must be other off-target effects for **4p** contributing to any observed cellular effects as well.

### 2.2. Assessment of Broad-Spectrum Anticancer Activities

#### 2.2.1. Inhibition of Cell Proliferation

**Figure 3:**
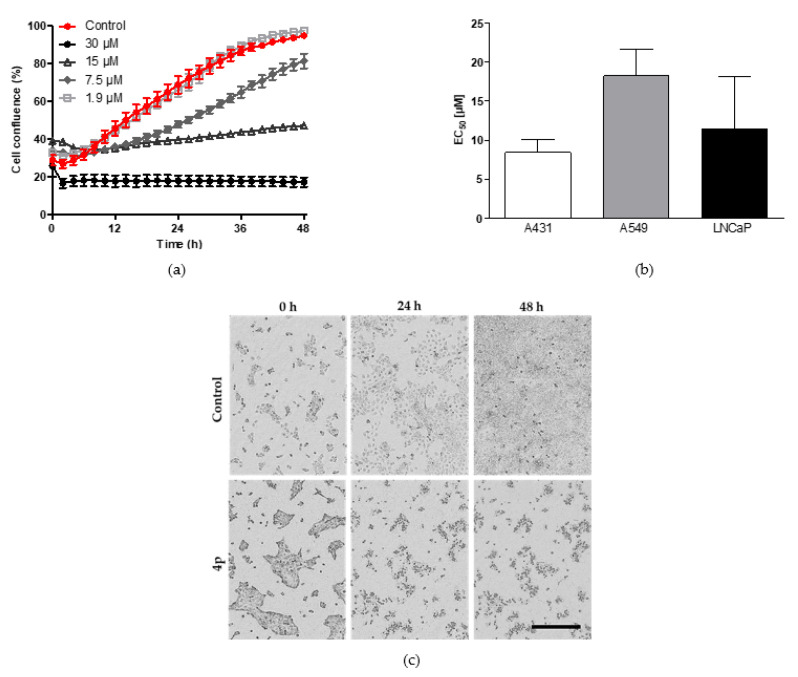
Investigation of the antiproliferative effect of **4p** in three different cancer cell lines. (**a**) Representative time-course of the percent confluence changes of A431 cells following treatment with **4p** at four different concentrations (1.9–30 µM) or vehicle (1% DMSO) for 48 h. (**b**) EC_50_ values of **4p** in A431, A549, and LNCaP cells. Data represent mean ± SD values from three independent experiments, each in triplicate. (**c**) Representative phase contrast images of A431 cells treated with 30 µM of **4p** or vehicle (1% DMSO control). Images were taken at three different time points: directly after treatment (0 h), at 24 h, and 48 h following treatment. Scale bar (400 µM) for all images is shown in black.

We previously showed the ability of **4p** to strongly reduce the proliferation rate of MCF7 breast cancer cell line using EdU-click assay suggesting its potential anticancer activity [33]. To comprehensively investigate the biological effects of **4p** on cancer cells, we examined the antiproliferative effects of this compound on cultured A431, A549, and LNCaP cells. Here, the confluence of cultured cells in 96-well plates was monitored for 48 h in a live cell imaging system (IncuCyte^®^, Sartorius, Michigan, MI, USA) following treatment with increasing concentrations of the compound (0.47–40 µM) and compared to vehicle-treated cells. As shown in Figure 3a, dose-dependent inhibition of the proliferation of A431 cells was observed, with the highest concentration applied (30 µM) leading to complete inhibition. Similar observations were made for treated A549 and LNCaP cells (Supplementary Materials, Figure S2). To determine the half-maximal effective concentration (EC_50_) values of **4p** in the tested cell lines, the area under the curve (AUC) values, determined for the different concentrations and normalized to control value, were plotted against the corresponding concentrations. Nonlinear regression analysis of the resulting dose-response plots was performed from which the EC_50_ values were obtained. The performed analysis demonstrated EC_50_ values of 8.4 ± 2.8 µM for A431 cells, 18.2 ± 6.0 µM for A549 cells, and 11.4 ± 6.8 µM for LNCaP cells (Figure 3b). Statistical analysis of the determined EC_50_ values by one-way ANOVA indicated no significant difference. Furthermore, we observed distinct morphological changes of all treated cells over the course of treatment (Figure 3c, Supplementary Materials, Figure S3).

Notably, we also analyzed the antiproliferative effect of **4p** on non-cancerous cells, namely Human Umbilical Vein Endothelial Cells (HUVECs), prepared and provided by the lab of Prof Gerke, Center for Molecular Biology of Inflammation, University of Münster. The antiproliferative EC_50_ of **4p** in HUVECs was determined in an analogous manner to those determined for the cancer cell lines A431, A549, and LNCaP and was found to be 10.6 ± 4.9 µM. This EC_50_ value of **4p** in HUVECs is in the same range as the EC_50_ values determined for A431, A549, and LNCaP cells. Although the obtained results may suggest high toxicity of **4p**, the different origin and sensitivity of HUVECs have to be considered before drawing conclusions on the toxicity of **4p**. This is supported by our observation that the reference CK2 inhibitor Silmitasertib [36], which was intensively evaluated for its safety in preclinical studies and has reached clinical trials [37,38], had an EC_50_ = 10.7 ± 1.5 µM on HUVECs in our experiments.

Taken together, these results indicate the dose-dependent and strong antiproliferative effects induced by **4p** in different types of cancer cells known to be overexpressing CK2. Thus, the observed antiproliferative effects of **4p** are likely attributed to cellular CK2 inhibition as derived from the cellular target engagement data shown in Figure 2b.

#### 2.2.2. Induction of Apoptosis

**Figure 4:**
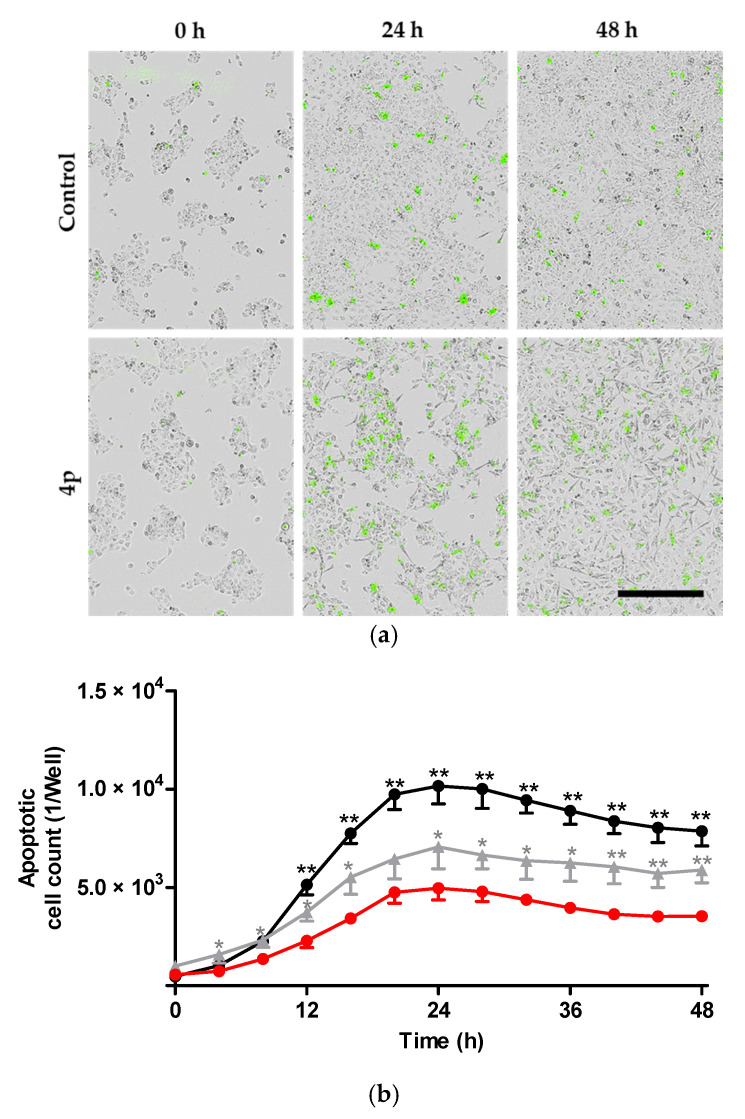
Induction of apoptosis by **4p** in cultured A431 cells. (**a**) Representative phase contrast images of A431 cells cultured in a 96-well plate and treated with 20 µM of **4p** or vehicle (1% DMSO control). All wells received IncuCyte^®^ caspase-3/7 green dye at a final concentration of 5 µM simultaneously. Images were taken at three different time points: directly after treatment (0 h), at 24 h, and 48 h following treatment. Green dots in the images represent apoptotic cells. Scale bar (400 µm) for all images is shown in black. (**b**) Monitoring of apoptotic events in cells treated with either 1 µM (gray) or 20 µM (black) of **4p**, compared to control (red) over a period of 48 h. Data represent mean ± SD from an experiment with three replicates. * *p* < 0.05, ** *p* < 0.01.

Next, we investigated whether apoptosis represents the main mechanism of A431 cell death following treatment with **4p**. For this purpose, an inert non-fluorescent caspase-3/7 substrate that freely crosses the cell membrane was added to the treated cultured cells. Activated caspase-3/7 in apoptotic cells can cleave the added substrate and release a green DNA-binding fluorescent dye (Figure 4a). Cells showing fluorescently-labeled nuclei were counted using IncuCyte^®^ S3 live-cell imaging system reflecting the extent of apoptotic events in wells of the treated cells. Here, the number of apoptotic cells showed a slight but statistically significant increase after treatment with 1 µM of **4p**. On the other hand, cells treated with 20 µM showed an almost two-fold increase in apoptotic events within the first 24 h following treatment as compared to vehicle control-treated cells (Figure 4b). These results reflect a high degree of activation of caspase 3/7 in response to cell death signals triggered by treatment of A431 cells with **4p**. In contrast, our previous observations in MCF7 cells revealed characteristic morphological features of apoptosis upon treatment with 100 µM, but not with 20 µM, of **4p** [33]. This discrepancy might be due to the fact that activated caspase-3/7 measured in the current study can accurately reflect early apoptotic events in cells, unlike the subjective examination of changes in nuclear size/shape.

#### 2.2.3. Inhibition of Cell Migration

**Figure 5:**
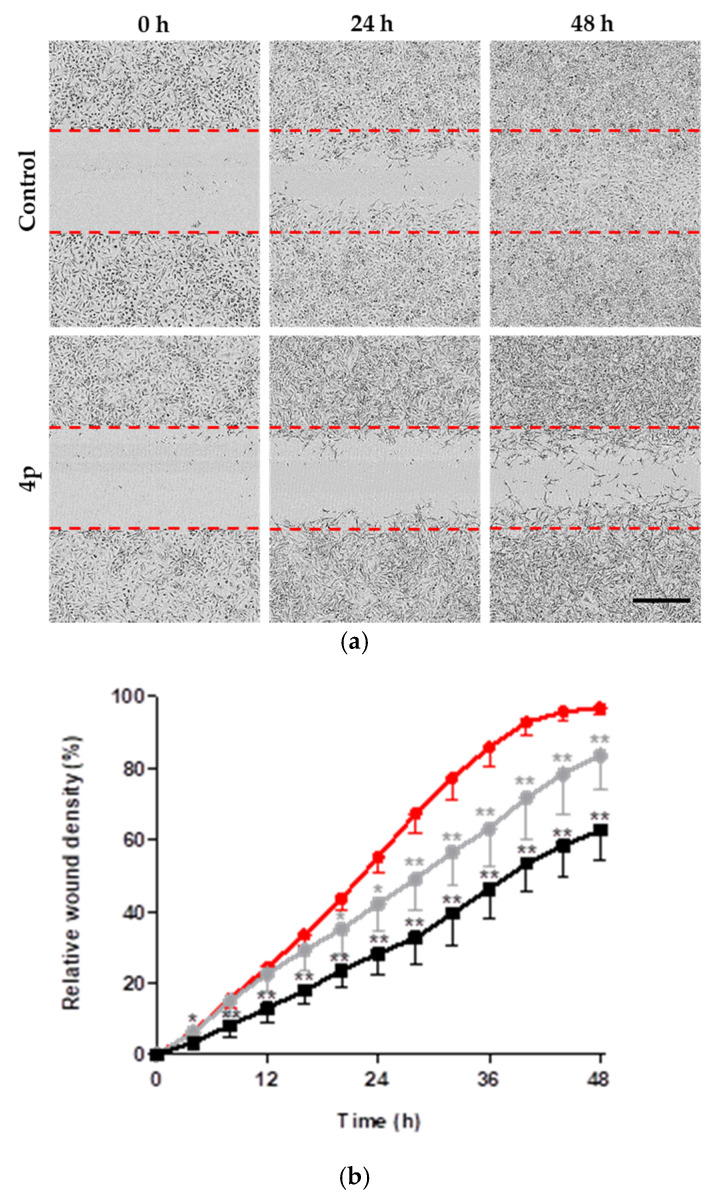
Inhibition of migration of A549 cells in a scratch wound assay. (**a**) Representative phase contrast images of scratch wounds in A459 cell layer grown to 100% confluence in 96-well imaging plates. Cells were treated with either vehicle (1% DMSO) or 10 µM **4p,** and the wound closure was examined directly after treatment (0 h), at 24 h, and 48 h following treatment. Dashed red lines represent the initial wound width. Scale bar (400 µM) for all images is shown in black. (**b**) Time-course of the relative wound density for cells treated with **4p** at 1 µM (gray) and 10 µM (black) or vehicle control (1% DMSO, red). Data represent mean ± SD from an experiment with three replicates. * *p* < 0.05, ** *p* < 0.01.

The potential of certain tumors to metastasize is closely associated with the ability of tumor cells to migrate and invade adjacent tissues. Upregulation of CK2 has been associated with the cytoskeletal reorganization and acquiring a migratory phenotype [39,40,41,42,43,44]. To assess the effect of **4p** on the migration capacity of A549 lung cancer cells, we examined the rate of cell migration following the induction of homogenous scratch wounds in 96-well plates. Monitoring of the induced scratch wounds for 48 h following treatment with **4p** at 10 µM revealed the impaired ability of A549 cells to migrate into the wound area while control cells that received 1% DMSO were able to completely seal the wound after 48 h (Figure 5a). To exclude the possibility that **4p**-induced impairment of cell migration at 10 µM is due to antiproliferative effect and not a direct anti-migratory effect, we examined the effect of **4p** at a concentration of 1 µM, which exhibits a very weak antiproliferative effect per se. Again, we could observe a statistically significant decrease in the percentage relative wound density (i.e., the ratio of the occupied area to the total area of the initial wound region) in the treated wells, compared to control (Figure 5b). These results suggest that **4p** has a direct inhibitory effect on cell migration, most likely due to inhibition of CK2 activity.

#### 2.2.4. Evaluation of Anticancer Effects in 3D Tumor Model

**Figure 6:**
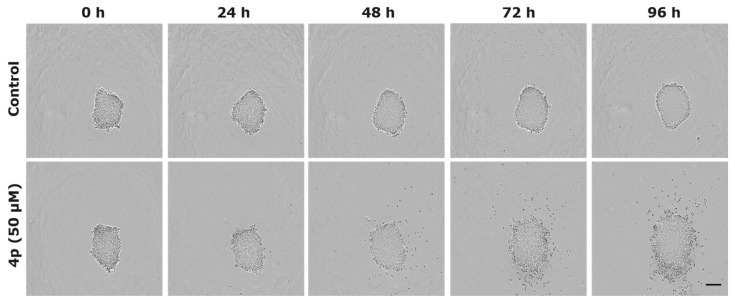
Effect of **4p** on the integrity of multicellular spheroids from A549 cell line. Representative phase-contrast images from time-lapse imaging of spheroids treated with either 50 µM of **4p** or vehicle control (0.5% DMSO). Increasing numbers of detached cells from the spheroid core start to appear at 48 h post-treatment with **4p**. The figure is representative of three independent experiments, each performed at least in duplicates. Scale bar (200 µM) for all images is shown in black.

Next, we investigated the effects of **4p** on the growth and integrity of multicellular tumor spheroids from A549 cell line. Spheroids have the ability to mimic the 3D structure of tumors and thereby serve as useful models for evaluating responses to anticancer drugs [45,46,47]. Here, spheroids were grown in 96-well plates for 72–96 h before treatment with 50 µM of **4p** or vehicle (0.5% DMSO). Monitoring of the treated spheroids revealed the development of a steadily growing diffuse outer layer of cells starting to appear 48 h after treatment with **4p** (Figure 6). An accurate estimation of the spheroid growth was not possible since this loosely attached layer to the spheroid core led to an overestimation of spheroid size. Each well received simultaneously 250 nM of IncuCyte^®^ cytotox red fluorescent dye to demonstrate the cytotoxic effect of **4p** on the treated spheroids. This dye did not perturb cell growth and yielded little intrinsic fluorescent signal in healthy cells. However, it permeated unhealthy cells with impaired plasma membrane integrity and bound DNA yielding a 100–1000-fold increase in fluorescence signals. Examination of the red fluorescence signals in the cultured A549 spheroids showed higher and uniformly distributed fluorescence signals in **4p** treated spheroids indicating a high number of dead cells over the exposed surface of the spheroids (Supplementary Materials, Figure S4). On the other hand, control spheroids demonstrated weak red fluorescence mostly localized to the necrotic core and not to the periphery of the spheroids (Supplementary Materials, Figure S4). Interestingly, a large proportion of the detached cells from the core of treated spheroids did not show red fluorescent signals, indicating that these cells were not dead. Notably, Virgone-Carlotta et al. [48] reported recently similar effects for the chemotherapeutic agent 5-flurouracil (5-FU). In that study, the ability of 5-FU to induce an anti cohesive effect on multicellular spheroids from the colorectal carcinoma cell lines HCT116 and SW48, following treatment with 10 µM of the drug, was described. The impaired spheroid cohesion in response to 5-FU was attributed to the ability of the drug to interfere with cell–cell and cell–matrix interaction in spheroids and was taken as a measure of the sensitivity of these cells to 5-FU. Similar to 5-FU, **4p** demonstrated a strong anti cohesive effect and resulted in detaching a mixture of dead and viable cells from the spheroid core. These viable detached cells seem to be resistant to the cytotoxic effects of **4p** and thereby simulating the drug-resistant cell subpopulations in tumor entities, which would therefore require multidrug treatment to eradicate them.

### 2.3. Investigation of In Vitro Pharmacokinetic Properties

#### 2.3.1. Cellular Uptake

**Figure 7:**
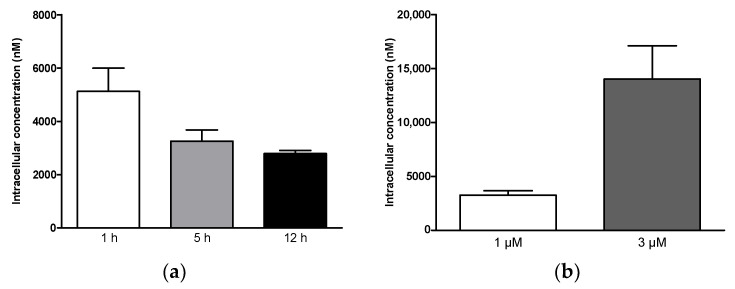
Uptake of **4p** in A431 cancer cell line. (**a**) Time-dependent changes in the intracellular concentration of **4p** in the presence of 1 µM of extracellular concentration of the compound. Cells were harvested at 1, 5, and 12 h post-treatment, counted, and lysed. (**b**) Concentration-dependent changes in the intracellular concentration of **4p** in the presence of 1 or 3 µM of extracellular concentration of the compound. Cells were harvested at 5 h post-treatment, counted, and lysed. In both (**a**,**b**), the concentration of **4p** in the soluble fraction of the lysate was determined via LC-MS/MS using matrix calibration. Data represent mean ± SD from an experiment with three replicates.

To better assess the suitability of **4p** as a potential anticancer drug, we investigated both the uptake in cancer cells and the metabolic stability of the compound. The changes in the intracellular concentration of **4p** in cultured A431 cancer cells were studied in both time- and concentration-dependent manners. First, the concentration of **4p** was determined in lysates from A431 cells using LC-MS/MS following preincubation of cells with 1 µM of the compound for 1, 5, and 12 h. Here, an intracellular concentration of approximately 5 µM was reached at 1 h post-treatment and continued to decline over the later time points to reach a value of 2.8 µM at 12 h post-treatment (Figure 7a). Furthermore, we observed approximately a four-fold increase in the intracellular concentration of **4p** (14 µM) upon increasing the extracellular concentration of the compound to 3 µM (Figure 7b). These results indicate the rapid uptake of the compound by A431 cells and suggest that the observed uptake was not due to a saturable transport process.

The observed higher intracellular concentration of **4p** as compared to the concentration of the compound in the cell culture medium indicates intracellular accumulation of the compound following its cellular uptake. Cellular retention of drugs has been previously described for kinase inhibitors, for example, imatinib, and is considered beneficial from a pharmacokinetic perspective [49]. A possible reason for this observed accumulation of **4p** might be the binding of the compound to cellular structures, e.g., plasma membrane [50], or inclusion in lipid droplets [51], leading to the retention of the substance in the treated cells. It would be important to investigate the mechanism(s) of cellular uptake as well as the subcellular distribution of **4p** to clarify this observation.

#### 2.3.2. In Vitro Metabolism Analysis

**Figure 8:**
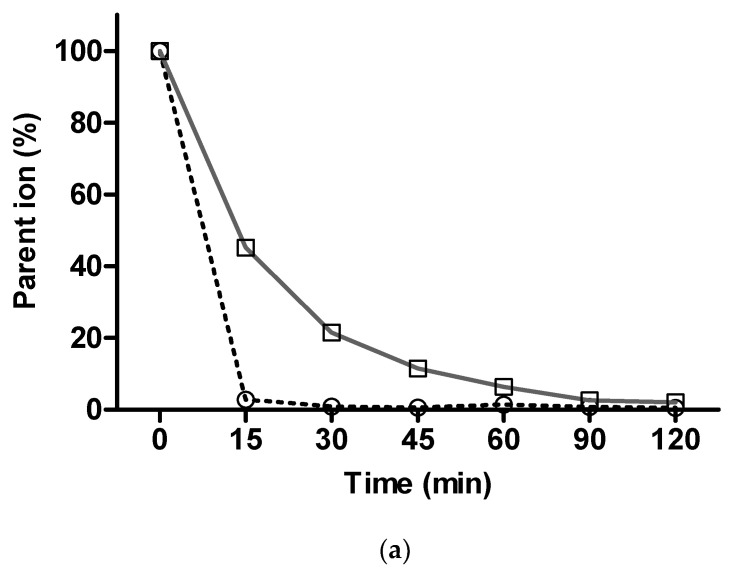

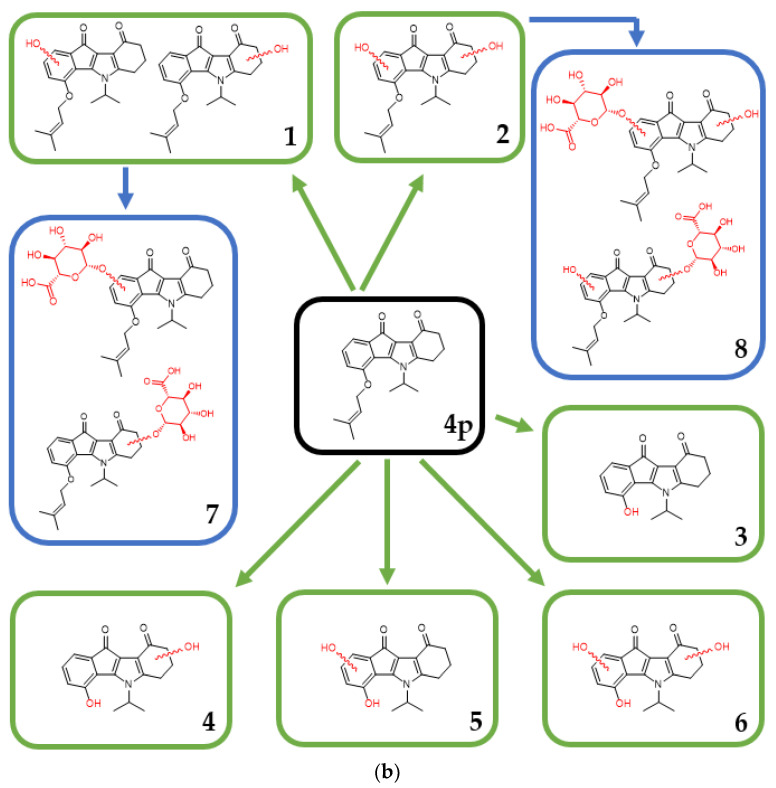
In vitro metabolism study of **4p**. (**a**) Metabolic stability profile of **4p** (dashed line) compared to that of imipramine (solid line). Both compounds were incubated with mouse liver microsomal fractions and NADPH for 120 min. The disappearance of the parent ion over time was measured in LC-MS/MS and normalized to the amount of parent ion at the start of the incubation (t = zero). (**b**) Schematic presentation of major identified metabolites of **4p** as elucidated from MS spectra following incubation with mouse liver microsomes and cofactors for phase I metabolism enzymes (green rectangles) and UDPGA to study glucuronidation (blue rectangles).

Next, the phase I metabolic stability of **4p** was assessed using imipramine, which is known to be metabolically labile per se [52], as a reference compound. Following incubation of 25 µM of each compound with mouse liver microsomal homogenate in the presence of cofactors, essential for phase I metabolic reactions, the percent parent ion was determined in LC-MS/MS at different time points, relative to the initial level of parent ion (t = 0). Here, the parent ion of **4p** almost completely disappeared after 15 min of incubation, while imipramine demonstrated a gradual decrease in the parent ion level up to the complete disappearance of its corresponding parent ion after 120 min (Figure 8a). These results indicate that **4p** was extensively metabolized in phase I reactions. Therefore, attempts were made to identify the major phase I as well as phase II metabolites of **4p**, whereby glucuronidation was analyzed as a representative for phase II metabolic reactions. Several mono- and dihydroxylated metabolites of **4p** (Figure 8(b1,2)) and its deprenylated derivative (Figure 8(b3–6)) could be identified, with the hydroxyl groups being added to ring A and/or ring D of the indeno[1,2-*b*]indole backbone. Furthermore, some of the hydroxylated metabolites were found to be glucuronidated in our experiments (Figure 8(b7,8)), suggesting that hydroxylation and glucuronidation may represent the major metabolic pathways for **4p**. These metabolites were not observed in control samples that did not contain NADPH as a cofactor for phase I metabolic enzymes and UDPGA as a cofactor for phase II metabolic enzymes in our study. This indicates the stability of **4p** in the absence of active metabolic processes.

Importantly, the approach taken to study the metabolism of **4p** in this study allowed the determination of the masses of the metabolites, but not the exact position of identical substituent as, for example, hydroxyl groups. In this analysis, it was also not possible to quantify the amount of the different metabolites obtained. Preliminary HPLC separation allowed, however, a distinction between the individual metabolites with similar masses, and thereby, we were able to determine the number of obtained **4p** metabolites (Supplementary Materials, Table S1, Figures S5–S11). It would be interesting in future studies to structurally characterize and test the CK2 inhibitory activity and cellular effects of these metabolites, particularly **1** and **2**, which were still bearing both the prenyloxy and isopropyl substitutions required for high CK2 inhibitory activity. Taken together, these results indicate the metabolic instability of **4p** and hints at the presence of active hydroxylated metabolites of the compound.

## 3. Materials and Methods

### 3.1. Chemicals

All chemicals were purchased in the highest available purity. **4p,** synthesized as described before in Gozzi et al. [33], was solved in dimethyl sulfoxide (DMSO) at a concentration of 10 mM and stored at −20 °C.

### 3.2. Cell Culture

Human epidermoid carcinoma cell line A431 and human lung carcinoma cell line A549 were cultured in DMEM high glucose medium (Life Technologies, Waltham, MA, USA) supplemented with 10% fetal calf serum (FCS). Both cell lines were kindly provided by Prof. Dr. Angelika Barnekow (Institute for Neuro- and Behavioural Biology, Westfälische Wilhelms-Universität, D-48149 Münster, Germany). Human prostate carcinoma cell line LNCaP, kindly provided by Prof. Dr. Claudia Götz (Department of Medical Biochemistry and Molecular Biology, Saarland University, D-66424 Homburg, Germany), was cultured in RPMI 1640 GlutaMax (Life Technologies, Waltham, MA, USA) supplemented with 10% FCS. All three cell lines were cultured at 37 °C in a humidified atmosphere of 5% CO_2_. Cells were cultured in 100 µL cell culture medium in 96-well plates and 2 mL in 6-well plates (Cellstar, Greiner, Darmstadt, Germany).

### 3.3. Determination of Intracellular CK2 Activity

The effect of **4p** on CK2 activity in cancer cells was determined using a modified protocol from the one described before by Schneider et al. [53]. Briefly, cells were cultured in 6-well plates for 48 h. Afterward, the medium was replaced with a 2 mL fresh cell culture medium containing 1 or 20 µM **4p** or vehicle control (1% DMSO) followed by 24 h incubation at 37 °C in a humidified atmosphere of 5% CO_2_. Subsequently, cells were harvested and suspended in lysis buffer (50 mM Tris/HCl (pH 7.5), 0.5% sodium desoxycholate, 0.15 mM NaCl, 1 mM Na_3_VO_4_, 0.5 mM NaF, 0.8 µM aprotinin, 10 µM pepstatin A, 20 µM leupeptin, 0.1 mM PMSF, 1 mM benzamidine, 1% Triton X-100). The protein concentration of the soluble fractions of the lysates was determined using a Bradford method (Roti-Quant, Roth, Karlsruhe, Germany). For each sample, 90 µg of protein was diluted in 80 µL kinase buffer (50 mM Tris/HCl pH 7.5, 100 mM NaCl, 10 mM MgCl_2_) and preincubated at 37 °C for 10 min. The reaction was started by mixing samples with 120 µL assay buffer (25 mM Tris/HCl (pH 8.5), 150 mM NaCl, 5 mM MgCl_2_, 190 µM FITC-RRRDDDSDDD, 100 µM ATP). Samples were incubated for 15 min at 37 °C before the kinase reaction was terminated by adding 5 mM EDTA and incubating samples on ice. Samples were analyzed via capillary electrophoresis (CE) as described in Gratz et al. 2010 [35] and detected using a laser-induced fluorescence (LIF) detector. Relative CK2 activity was calculated in each sample using the ratio of AUC values of detected product and substrate peaks and normalized to that of vehicle control (1% DMSO). The latter was set as 100% activity.

### 3.4. IncuCyte^®^ Cell Proliferation Assay

A431, A549, and LNCaP cells were cultured in 96-well plates to a confluence of approximately 30%. Then, the culture medium in each well was removed and replaced with 100 µL fresh medium containing vehicle control (1% DMSO) or **4p** in at least eight different concentrations. Afterward, cells were placed in an IncuCyte^®^ S3 live-cell imaging system (Sartorius, Ann Arbor, MI, USA) at 37 °C in a humidified atmosphere of 5% CO_2_ for a total period of 48 h. Microscopic phase contrast images of all wells were taken at 2 h intervals with a ten-fold lens (four images per well). The percent cell confluence in each well was determined for all time points using the IncuCyte S3 2017A software. GraphPad Prism 5 software (GraphPad software, La Jolla, CA, USA) was used to determine the area under the obtained growth curves (AUC). The percent confluence at the beginning of each growth curve was set as the baseline, respectively. The AUC values of the obtained curves were normalized to the corresponding AUC of the vehicle control and plotted against the logarithmic values of the applied **4p** concentrations. A nonlinear regression analysis was performed to determine an EC_50_ value for each cell line using GraphPad Prims 5 software.

### 3.5. IncuCyte^®^ Cell Apoptosis Assay

A431 cells were seeded into 96-well plates and cultured until a confluence of 30–40% was reached. Subsequently, the culture medium in the wells was removed and replaced with 100 µL fresh medium containing either 1 or 20 µM of **4p** while control wells received 1% DMSO. The added solution to each well contained 5 µM of a green caspase 3/7 apoptosis reagent (Sartorius, Ann Arbor, MI, USA). This fluorescent dye is bound to a quencher via a peptide chain. The enzymes caspase 3 and 7, which are only active during the induction of apoptosis in eukaryotic cells, are able to cleave this peptide chain. Subsequently, the dye intercalates into the DNA in the nucleus of the apoptotic cells and its fluorescence signal is detectable. After treatment, cells were stored in an IncuCyte^®^ S3 live-cell imaging system for 48 h at 37 °C and 5% CO_2_. Fluorescence microscopy images of the wells were taken every 2 h with a ten-fold lens (four images per well) using the green fluorescence channel of the IncuCyte^®^ S3 live-cell imaging system (excitation 440–480 nm, emission 504–544 nm). The number of apoptotic cells was derived from the number of green fluorescent events determined with the IncuCyte^®^ 2017A Rev2 software.

### 3.6. IncuCyte^®^ Scratch Wound Migration Assay

Image-lock plates with 96-wells (Sartorius, Ann Arbor, MI, USA) were coated with a collagen-I solution (300 µg/mL) in 0.02 M acetic acid to study the migration of A549 lung carcinoma cells. Subsequently, 2.5 × 10^4^ A549 cells per well were seeded into the prepared image-lock plates. The cells were cultured at 37 °C and 5% CO_2_ until 100% confluence was achieved. Next, a wound was created in the center of each well using a WoundMaker^TM^ (Sartorius, Ann Arbor, MI, USA) followed by washing the wells twice with 120 µL of the pre-warmed medium. Wells then received 100 µL of fresh medium containing either vehicle (1% DMSO) or **4p** at two different concentrations (1 and 10 µM). Microscopic phase contrast images were then taken at 2 h intervals over a 48-h period in IncuCyte^®^ S3 live cell imaging system and analyzed using the IncuCyte^®^ 2017A Rev2 software to calculate the relative wound density in each well at the corresponding time points.

### 3.7. IncuCyte^®^ 3D Spheroid Imaging

Multicellular spheroids from the A549 cell line were initiated and cultured according to the protocol of Friedrich et al. [54] to evaluate the effects of **4p** on a 3D tumor model. Briefly, 96-well plates were coated with 45 µL of sterile 1% agarose solution, left to solidify for 10 min followed by seeding of 2.0 × 10^3^ cells/well in 200 µL of culture medium. Cells were then incubated at 37 °C in a humidified atmosphere of 5% CO_2_ to allow spheroid initiation and left to grow for 72–96 h. On the day of treatment, 125 µL of culture medium were carefully removed from each well followed by the addition of 75 µL of fresh medium containing 2× of the desired final concentrations of **4p** (50 µM) or vehicle (0.5% DMSO) and IncuCyte^®^ cytotox red fluorescent dye (250 nM) (Sartorius, Ann Arbor, MI, USA). Cultured spheroids were monitored for 96 h using IncuCyte^®^ S3 live-cell imaging system maintained at 37 °C and 5% CO_2_ using both phase contrast and red fluorescence channels.

### 3.8. Uptake Analysis

Cells were seeded in 6-well plates at a density of 5.0 × 10^5^ per well and cultured for 48 h at 37 °C and 5% CO_2_. Subsequently, the complete medium in each well was removed and replaced with a 2 mL fresh medium containing 1 or 3 µM **4p**. Cells were incubated with **4p** for 1, 5, or 12 h. Then, cells were washed with PBS and detached from the wells using trypsin/EDTA. The obtained suspensions of cells were then centrifuged for 5 min at 700× *g* and 4 °C. The number of cells in the resulting pellet of each sample was determined using an automated cell counter (Scepter^®^, Merck, Darmstadt, Germany). After washing with PBS, cells were lysed in 100 µL homogenization buffer (50 mM Tris/HCl (pH 7.5), 1 mM EDTA, 1 mM Na_3_VO_4_, 0.5 mM NaF, 0.1 mM PMSF, 1 mM benzamidine, 1% Triton X-100). Proteins were precipitated by adding 500 µL of ice-cold acetonitrile/methanol 1/1 (*v*/*v*) and separated by centrifugation for 30 min at 4 °C and 20,000× *g*. Five hundred microliters of the supernatants were evaporated to dryness using a vacuum centrifuge (Christ) for 1.5 h at 50 °C under full vacuum and suspended in 100 µL acetonitrile/H_2_O 1/1 (*v*/*v*). Samples were then transferred to micro inlets for HPLC vials (BGB, Rheinfelden, Germany) and analyzed by high-performance liquid chromatography coupled with detection via triple quadrupole mass spectrometry (HPLC-MS/MS, QTrap^®^ 6500^+^, AB Sciex, Darmstadt, Germany).

External matrix calibration was performed for the quantitative determination of the intracellular concentration of **4p**. Intracellular concentrations were calculated as described in Rahnel et al. [55] with the following equation.

(1) $$c_{x}=\frac{A_{x}-\;b}{a}{\;\times \;V}_{\mathrm{final}}\;\times \frac{1}{N_{\mathrm{cells}}\;\times \;\frac{4}{3}\;\pi \;\times \;{\left(\frac{d_{\mathrm{cells}}}{2\;\times \;1000}\right)}^{3}}\;\times \;1.01$$

where c_x_ is the intracellular concentration of **4p**, A_x_ is the area of the analyte signal in the respective chromatogram, b is the ordinate, and a is the slope of the calibration line, V_final_ is the final sample volume, N_cells_ is the cell number of the sample and d_cells_ is the mean diameter of the cells in the sample. The factor 1.01 takes into account the sample volume taken for cell count determination.

### 3.9. In Vitro Metabolism Analysis

The metabolic stability determination and the identification of metabolites of **4p** were performed using a method previously described by Börgel et al. [56]. For the analysis of the metabolic stability of **4p**, 25 µM of the substance were dissolved in PBS supplemented with 12.5 mM MgCl_2_ and 0.5 mg/mL NADPH. Next, mouse liver microsomes were added to the reaction (0.5 mg/mL protein). Samples were incubated 15–120 min at 37 °C and 900 rpm in a thermomixer (Eppendorf, Hamburg, Germany). The reaction was terminated by adding 400 µL ice-cold acetonitrile/H_2_O 1/1 (*v*/*v*) to each sample. Samples were centrifuged for 10 min at 4 °C and 20,000× *g*. Supernatants were analyzed via HPLC-MS/MS.

Metabolites of **4p** were identified as described above. In contrast to the metabolic stability analysis, 0.5 mg/mL UDPGA was used beside NADPH to analyze glucuronidation representative for phase II metabolism. Samples were analyzed with a high-resolution LC-qToF-MS system (Bruker Daltonics, Billerica, MA, USA). The identification of metabolites of **4p** was performed by determining molecular formulas from the exact masses of each metabolite using Data Analysis software (Bruker Daltonics, Billerica, MA, USA).

### 3.10. HPLC Analysis

For the cellular uptake and metabolism analysis, samples were investigated using a Nexera X2 high-performance liquid chromatography (HPLC) system (Shimadzu, Kyoto, Japan) coupled with a triple quadrupole mass spectrometer (QTrap^®^ 6500^+^, AB Sciex, Darmstadt, Germany). A RP18 column (XSelect HSS T3, 100 mm × 2.10 mm, 2.5 µm, Waters, Milford, MA, USA) was used as the stationary phase here, which was tempered to 40 °C. A solvent gradient of acetonitrile/H_2_O 10/90 (*v*/*v*) + 0.1% (*v*/*v*) formic acid (A) and acetonitrile/H_2_O 90/10 (*v*/*v*) + 0.1% (*v*/*v*) formic acid (B) served as the mobile phase. The flow rate was set to 0.3 mL/min. The gradient was run as follows: 0% B to 100% B: 0–7 min; 100% B 7–9 min; 100% B to 0% B: 9–9.5 min; 0% B: 9.5–15 min. The injection volume of the samples was 5 µL. Analytes were ionized via electrospray ionization (ESI) and detected in multiple reaction monitoring (MRM) mode. MS parameters were optimized on a compound-specific basis.

The metabolite identification was performed using an Ultimate 3000 RS HPLC system (Dionex, Sunnyvale, CA, USA) in combination with a high-resolution micrOTOF-Q II mass spectrometer (Bruker Daltonics, Billerica, MA, USA). The stationary phase and the solvent composition were as described above. The solvent gradient was run as follows: 0% B: 0–1 min; 0% B to 100% B 1–12 min; 100% B: 12–14.5 min; 100% B to 0% B: 14.5–15 min; 0% B: 15–18 min. The flow rate was set at 0.4 mL/min. The injection volume was 10 µL. Analyte ionization was performed via ESI. For metabolite identification, exact masses of ions with *m*/*z* 70–700 were determined.

### 3.11. Statistical Analysis

Levels of significance for comparison of two treatment groups in Figure 2b or control and each treatment group in Figure 4b and Figure 5b were determined using an unpaired Student’s *t*-test. Comparison of the EC_50_ values determined in the three cell lines studied (Figure 3b) was performed by one-way ANOVA. The *p*-values are indicated in the corresponding figures as follows: * *p* < 0.05, ** *p* <0.01. All statistical analyses were performed using GraphPad Prism 5 Software.

## 4. Conclusions

In previous work, we identified the prenyloxy-substituted indeno[1,2-*b*]indole, known as **4p**, as a very potent inhibitor of CK2 holoenzyme using in vitro kinase activity assay. In the present work, we provide evidence for the intracellular inhibition of CK2 in three different in vitro cancer models indicating the cellular target engagement of **4p**. Using live-cell imaging, we thoroughly investigated the biological effects of **4p** on several CK2-mediated cellular processes associated with cancer in 2D and 3D cell cultures. **4p** shows convincing antiproliferative, anti-migratory, and apoptosis-inducing effects and seems to disrupt cell/cell and cell/matrix interactions as indicated by the impaired cohesion of tumor spheroids. The strong anticancer effects of **4p** suggest high intracellular concentrations of the compound, which was confirmed in the present study. We further show that **4p** is extensively metabolized, giving rise to several hydroxylated and/or deprenylated metabolites, which can be glucuronidated, posing a challenge to improve the drug-like properties of the compound. The evaluation of the CK2 inhibitory potential and cellular activities of these metabolites should be considered for future work.

## Data Availability

The data presented in this study are available in the article and Supplementary Materials.

## Supplementary Materials

The following are available online https://www.mdpi.com/article/10.3390/ph14060542/s1, Figure S1: Representative electropherogram of capillary electrophoresis-based measurements of cellular CK2 kinase activity, Figure S2: Representative time-course of the percent confluence changes of (a) A549 and (b) LNCaP cells following treatment with **4p**, Figure S3: Representative phase contrast images of (a) A549 and (b) LNCaP cells treated with **4p**, Figure S4: Representative red fluorescence channel images (a) and overlay with phase contrast images (b) of A549 spheroids showing the cytotoxic effect of **4p**, Table S1 and Figures S5–S11: Identification of **4p** metabolites.
