# Supplementary material for: Broad-Spectrum Anticancer Activity and Pharmacokinetic Properties of a Prenyloxy-Substituted Indeno[1,2-b]indole Derivative, Discovered as CK2 Inhibitor
Source: Pharmaceuticals (Basel). 2021 Jun 5;14(6):542. doi: 10.3390/ph14060542 (PMC8226678; doi:10.3390/ph14060542)
Supplement: Supplementary file 1 [file pharmaceuticals-14-00542-s001.zip › pharmaceuticals-1221852-supplementary.pdf]

Supplementary Materials

Figure S1.

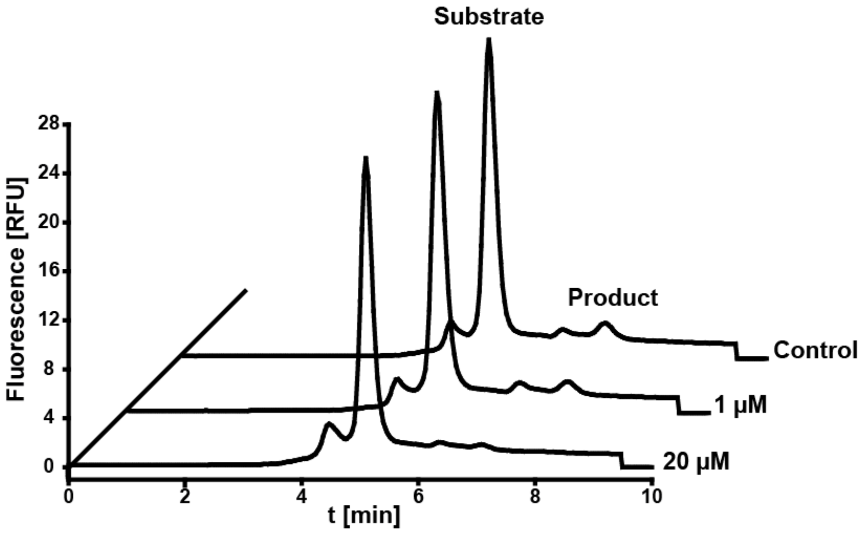

Figure S1. Representative electropherogram of capillary electrophoresis-based measurements of cellular CK2 kinase activity. Peaks of the fluorescently labelled CK2 substrate (FITC-RRRDDDSDD-NH2) and the corresponding peak of the phosphorylated product were detected in the soluble fractions of lysates from A431 cells using a laser-induced fluorescence (LIF) detector. Before lysis, cells were treated with 1 or 20 µM of **4p** and control cells were treated with 1% DMSO.

Figure S2.

(a)

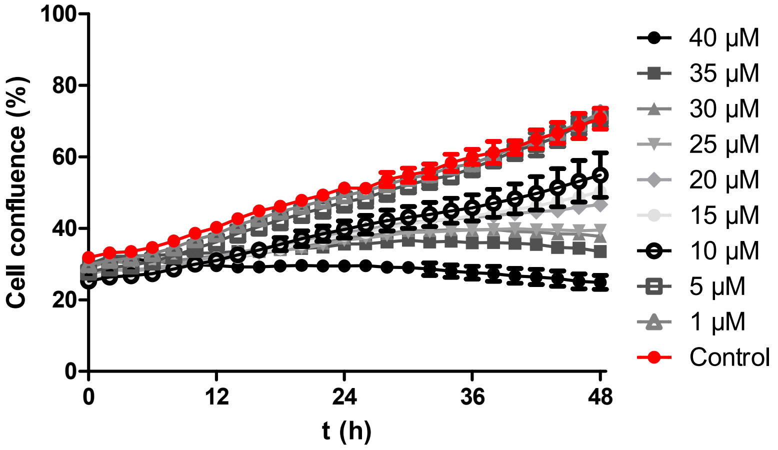

(b)

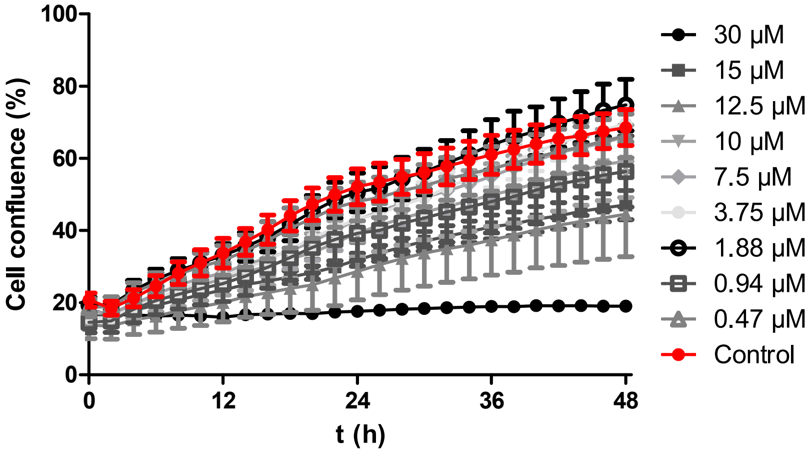

Figure S2. Representative time-course of the % confluence changes of (a) A549 and (b) LNCaP cells following treatment with **4p** at nine different concentrations (1-40 µM and 0.47-30 µM for A549 and LNCaP cells, respectively). In both (a) and (b) control wells received 1% DMSO. Cells were monitored for 48 h following treatment using IncuCyte® S3 live cell imaging system and % confluence values were analyzed using the IncuCyte® S3 2017A software.

Figure S3.

(a)

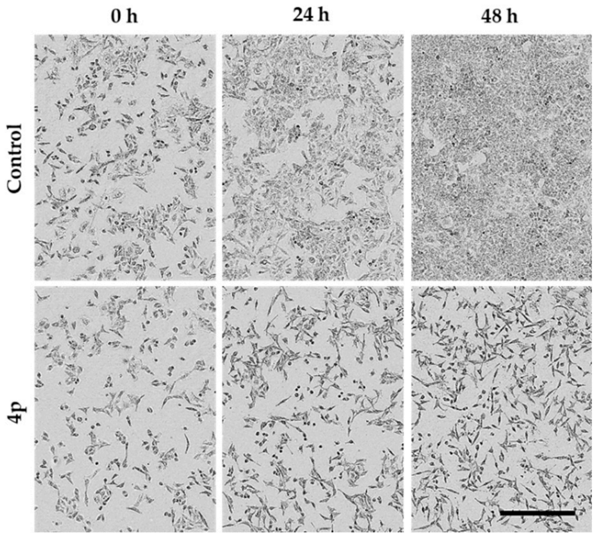

(b)

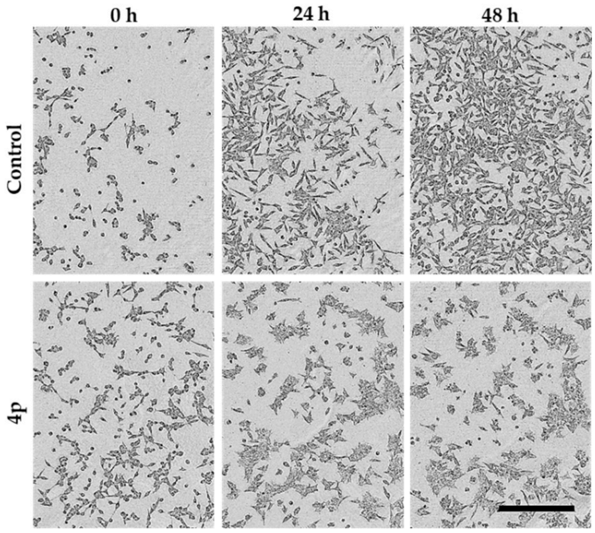

Figure S3. Representative phase contrast images of (a) A549 and (b) LNCaP cells treated with 40 μM and 30 μM of **4p**, respectively or vehicle (1% DMSO control). Images were taken at three different time points; directly after treatment (0 h), at 24 h and at 48 h following treatment. Images were obtained using the IncuCyte® live cell imager. Scale bar (400 μm) for all images is shown in black.

Figure S4.

(a)

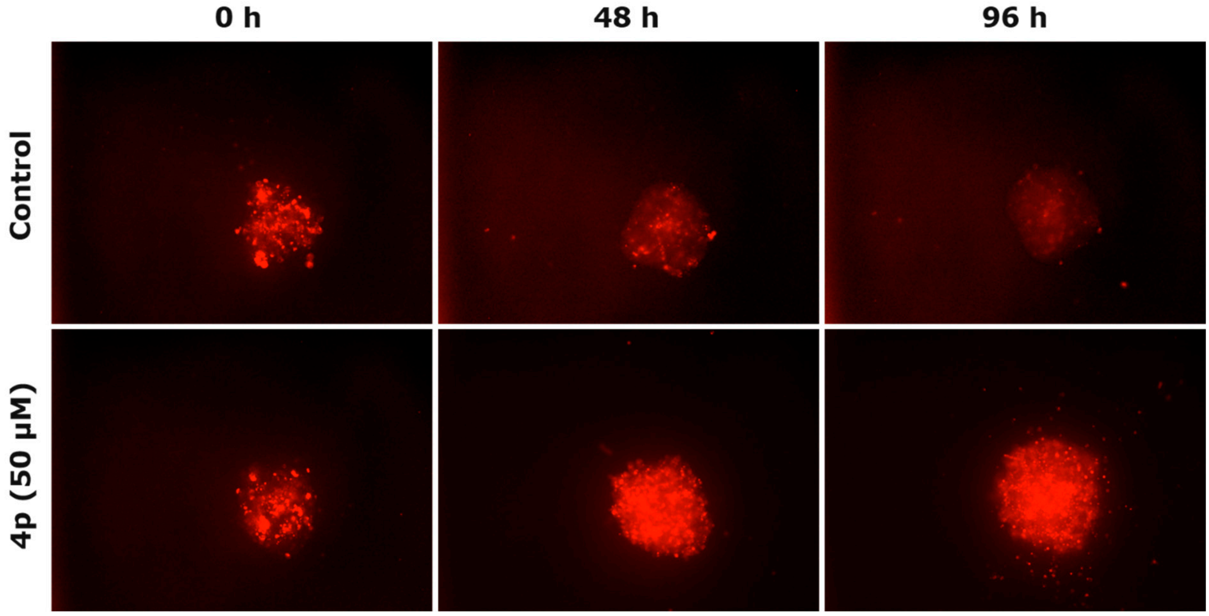

(b)

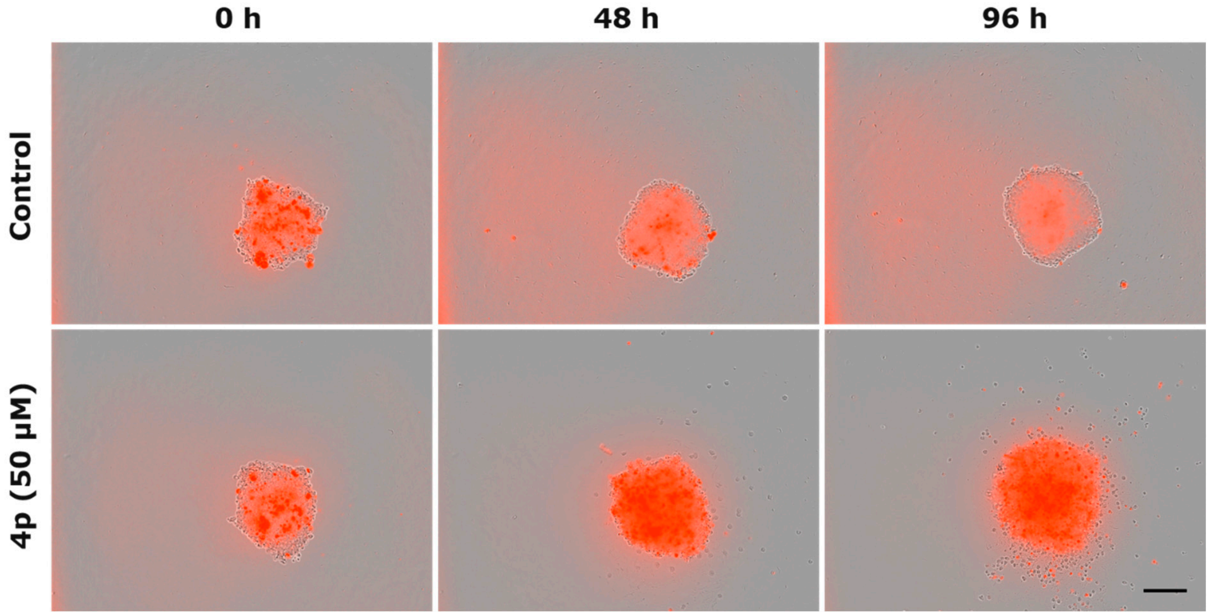

Figure S4. Representative red fluorescence channel images (a) and overlay with phase contrast images (b) of A549 spheroids showing the cytotoxic effect of **4p**. Spheroids of A549 cell line were treated with 50 µM of **4p** or vehicle (1% DMSO control) and received simultaneously 250 nM of Incucyte® cytotox red fluorescent dye. Images were taken at three different time points; directly after treatment (0 h), at 48 h and at 96 h following treatment. Images were obtained using the IncuCyte® live cell imager. Scale bar (200 µm) for all images is shown in black.

Table S1. Identified phase I and phase II metabolites of **4p**

Phase	*m*/*z*	Molecular formula	Metabolite	Number of identified species
I	364.1800	$C_{23}H_{26}NO_3$	Parent ion	1
	380.1844	$C_{23}H_{26}NO_4$	Monohydroxylated	5
	396.1798	$C_{23}H_{26}NO_5$	Dihydroxylated	6
	296.1273	$C_{18}H_{18}NO_3$	Deprenylated	1
	312.1229	$C_{18}H_{18}NO_4$	Monohydroxylated + deprenylated	1
	328.1183	$C_{18}H_{18}NO_5$	Dihydroxylated + deprenylated	1
I + II	556.2168	$C_{29}H_{34}NO_{10}$	Monohydroxylated + glucuronidated	5
	572.2137	$C_{29}H_{34}NO_{11}$	Dihydroxylated + glucuronidated	2

Figure S5.

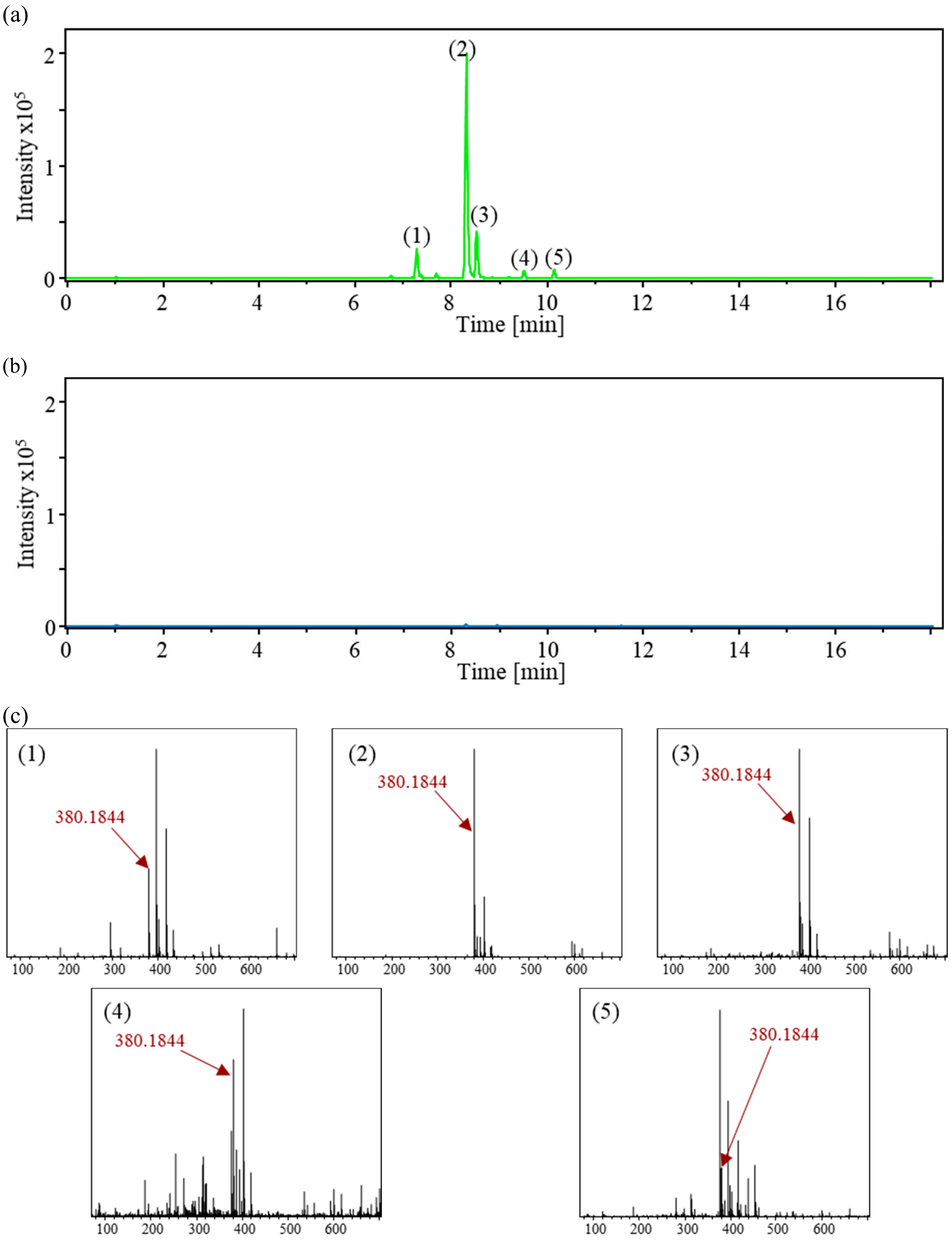

Figure S5. Identification of monohydroxylated metabolites of **4p**. Based peak chromatograms (m/z 380.1844) of monohydroxylated metabolites of **4p** (Figure 8b 1) of samples incubated with mouse liver microsomal homogenates for 90 min at 37 °C in the presence (a) or absence (b) of NADPH as cofactor for phase I metabolism enzymes. (c) Mass spectra of monohydroxylated metabolite species shown in (a). Metabolites were identified from these spectra via expected molecular formulas from Data Analysis Software (Bruker Daltonics) with errors below 10 ppm.

Figure S6.

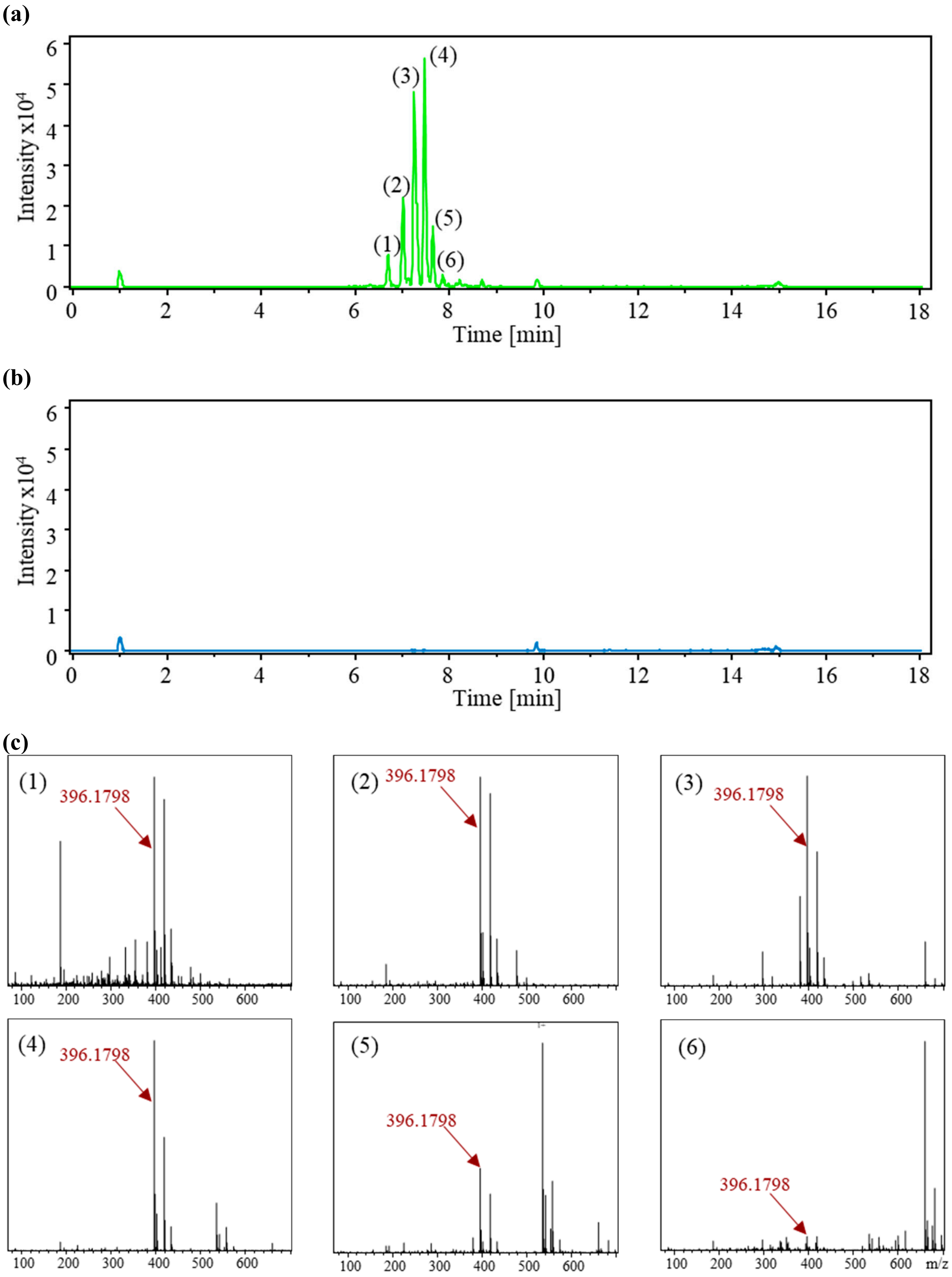

Figure S6. Identification of dihydroxylated metabolites of **4p**. Based peak chromatograms (m/z 396.1798) of dihydroxylated metabolites of **4p** (Figure 8b 2) of samples incubated with mouse liver microsomal homogenates for 90 min at 37 °C in the presence (a) or absence (b) of NADPH as cofactor for phase I metabolism enzymes. (c) Mass spectra of dihydroxylated metabolite species shown in (a). Metabolites were identified from these spectra via expected molecular formulas from Data Analysis Software (Bruker Daltonics) with errors below 10 ppm.

Figure S7.

(a)

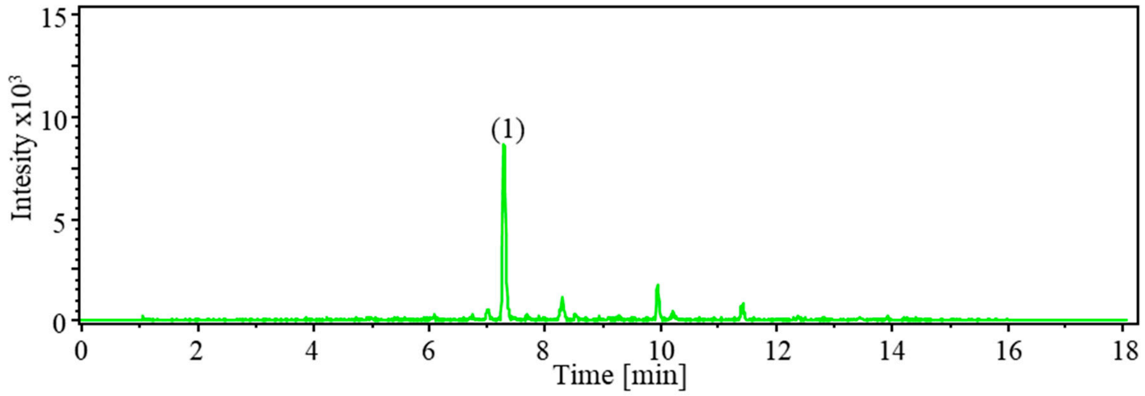

(b)

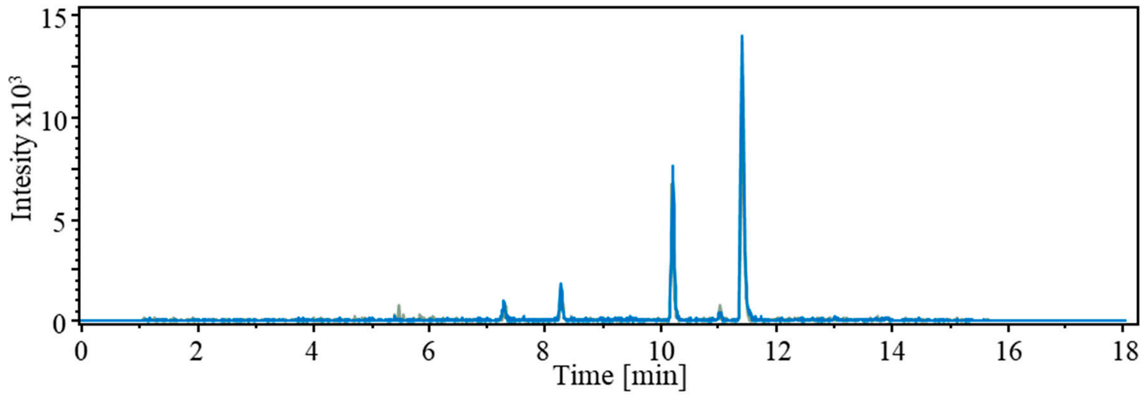

(c)

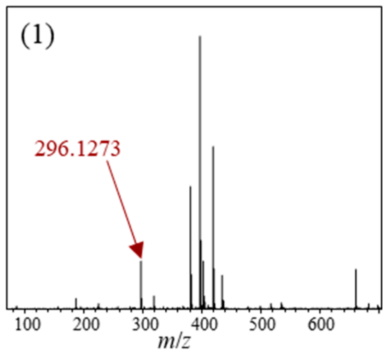

Figure S7. Identification of deprenylated metabolites of **4p**. Based peak chromatograms (m/z 296.1273) of deprenylated metabolites of **4p** (Figure 8b 3) of samples incubated with mouse liver microsomal homogenates for 90 min at 37 °C in the presence (a) or absence (b) of NADPH as cofactor for phase I metabolism enzymes. (c) Mass spectrum of deprenylated metabolite species shown in (a). The metabolite was identified from this spectrum via expected molecular formulas from Data Analysis Software (Bruker Daltonics) with errors below 10 ppm.

Figure S8.

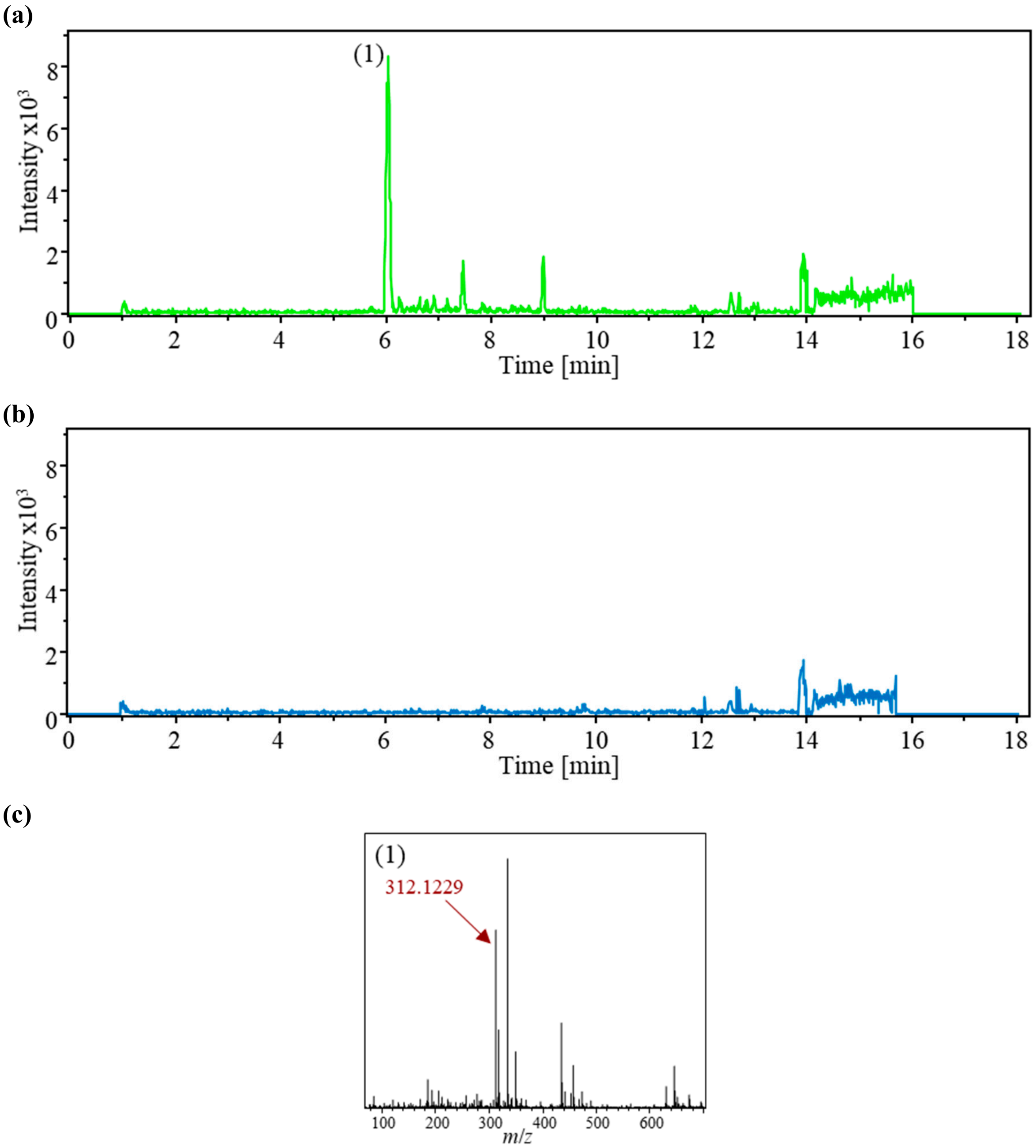

Figure S8. Identification of monohydroxylated and deprenylated metabolites of **4p**. Based peak chromatograms (m/z 312.1229) of monohydroxylated and deprenylated metabolites of **4p** (Figure 8b 4&5) of samples incubated with mouse liver microsomal homogenates for 90 min at 37 °C in the presence (a) or absence (b) of NADPH as cofactor for phase I metabolism enzymes. (c) Mass spectrum of monohydroxylated and deprenylated metabolite species shown in (a). The metabolite was identified from this spectrum via expected molecular formulas from Data Analysis Software (Bruker Daltonics) with errors below 10 ppm.

Figure S9.

(a)

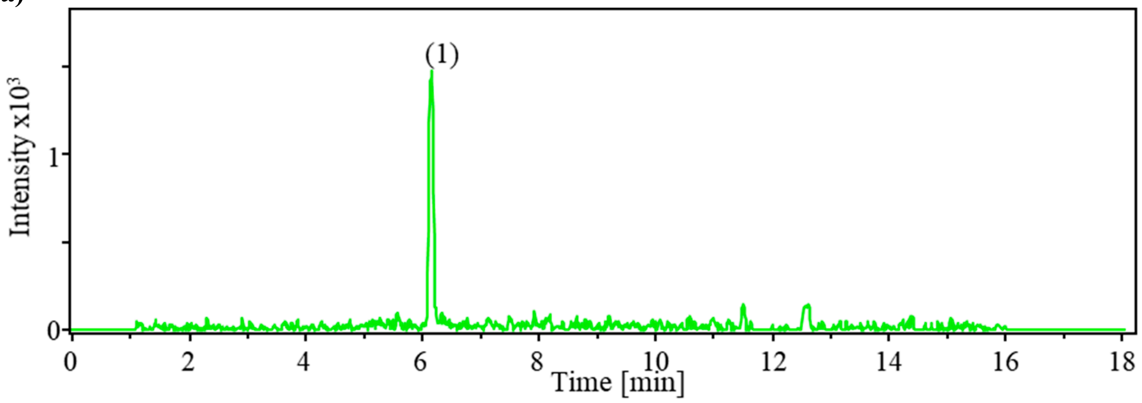

(b)

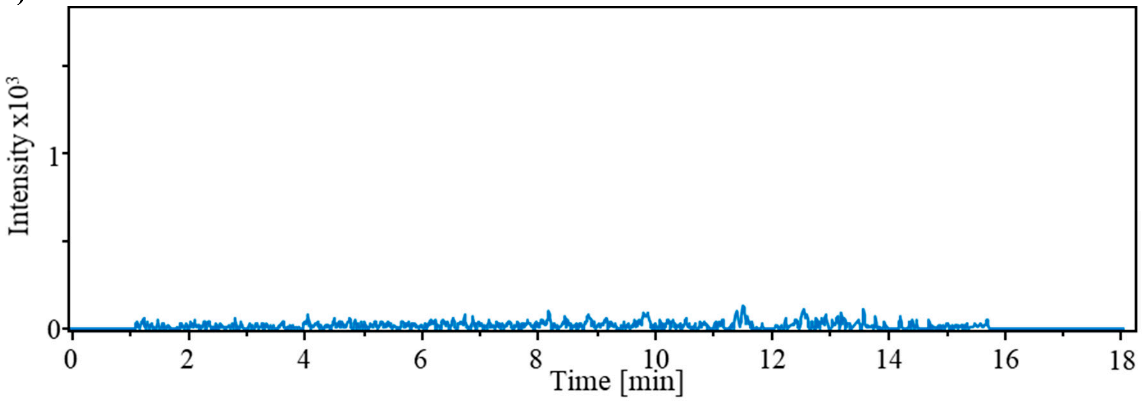

(c)

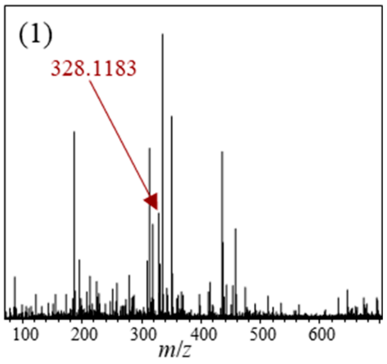

Figure S9. Identification of dihydroxylated and deprenylated metabolites of **4p**. Based peak chromatograms (m/z 328.1183) of dihydroxylated and deprenylated metabolites of **4p** (Figure 8b 6) of samples incubated with mouse liver microsomal homogenates for 90 min at 37 °C in the presence (a) or absence (b) of NADPH as cofactor for phase I metabolism enzymes. (c) Mass spectrum of dihydroxylated and deprenylated metabolite species shown in (A). The metabolite was identified from this spectrum via expected molecular formulas from Data Analysis Software (Bruker Daltonics) with errors below 10 ppm.

Figure S10.

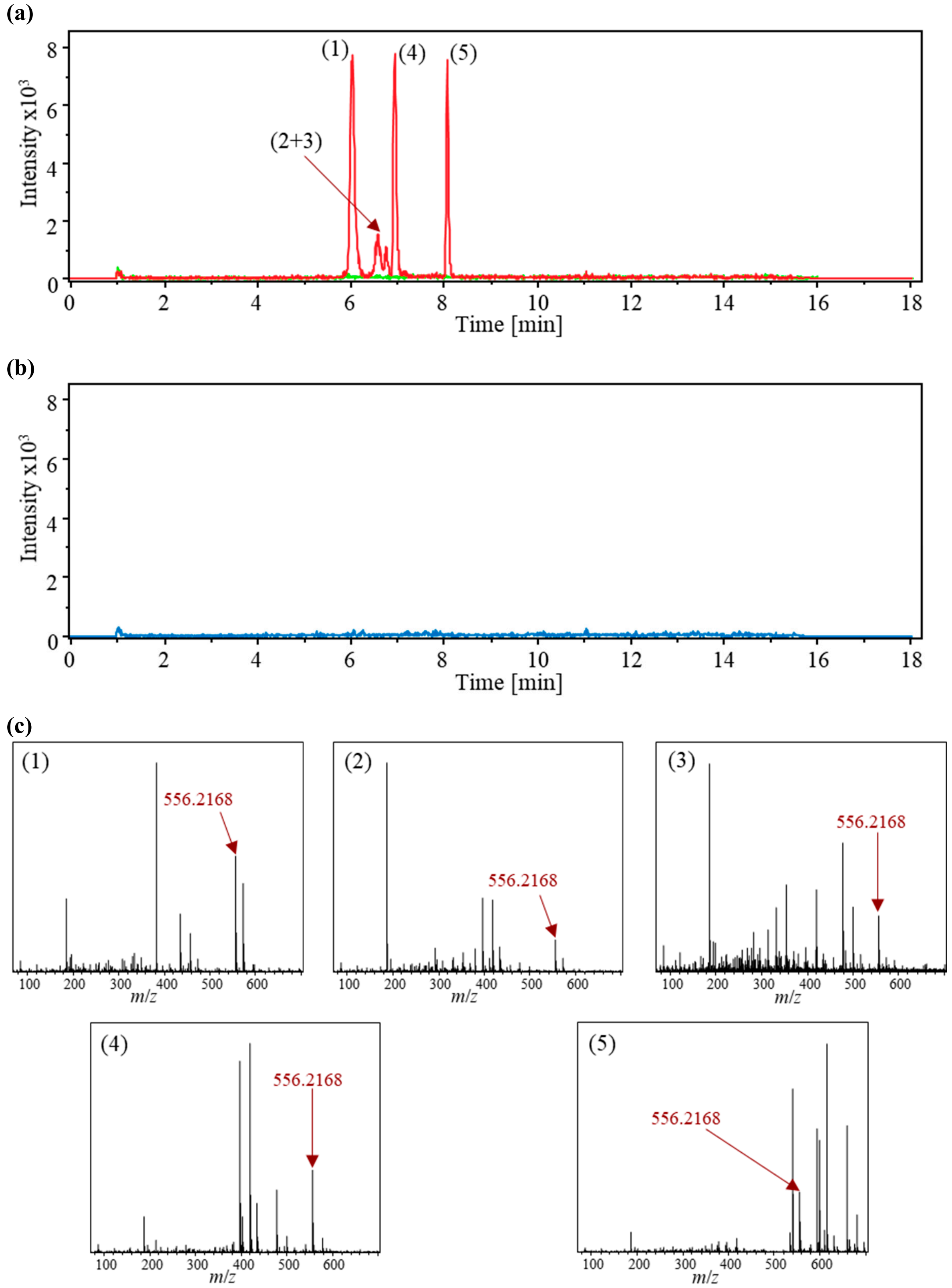

Figure S10. Identification of glucuronidated metabolites of **4p**. (a) Based peak chromatograms (m/z 556.2168) of glucuronidated metabolites of **4p** (Figure 8b 7) of samples incubated with mouse liver microsomal homogenates for 90 min at 37 °C in the presence of NADPH (green) and NADPH + UPGA (red) as cofactors for phase I and phase II metabolism enzymes. (b) Based peak chromatogram of **4p** treated analogously to (a) but without cofactors. (c) Mass spectra of glucuronidated metabolite species shown in (a). Metabolites were identified from these spectra via expected molecular formulas from Data Analysis Software (Bruker Daltonics) with errors below 10 ppm.

Figure S11.

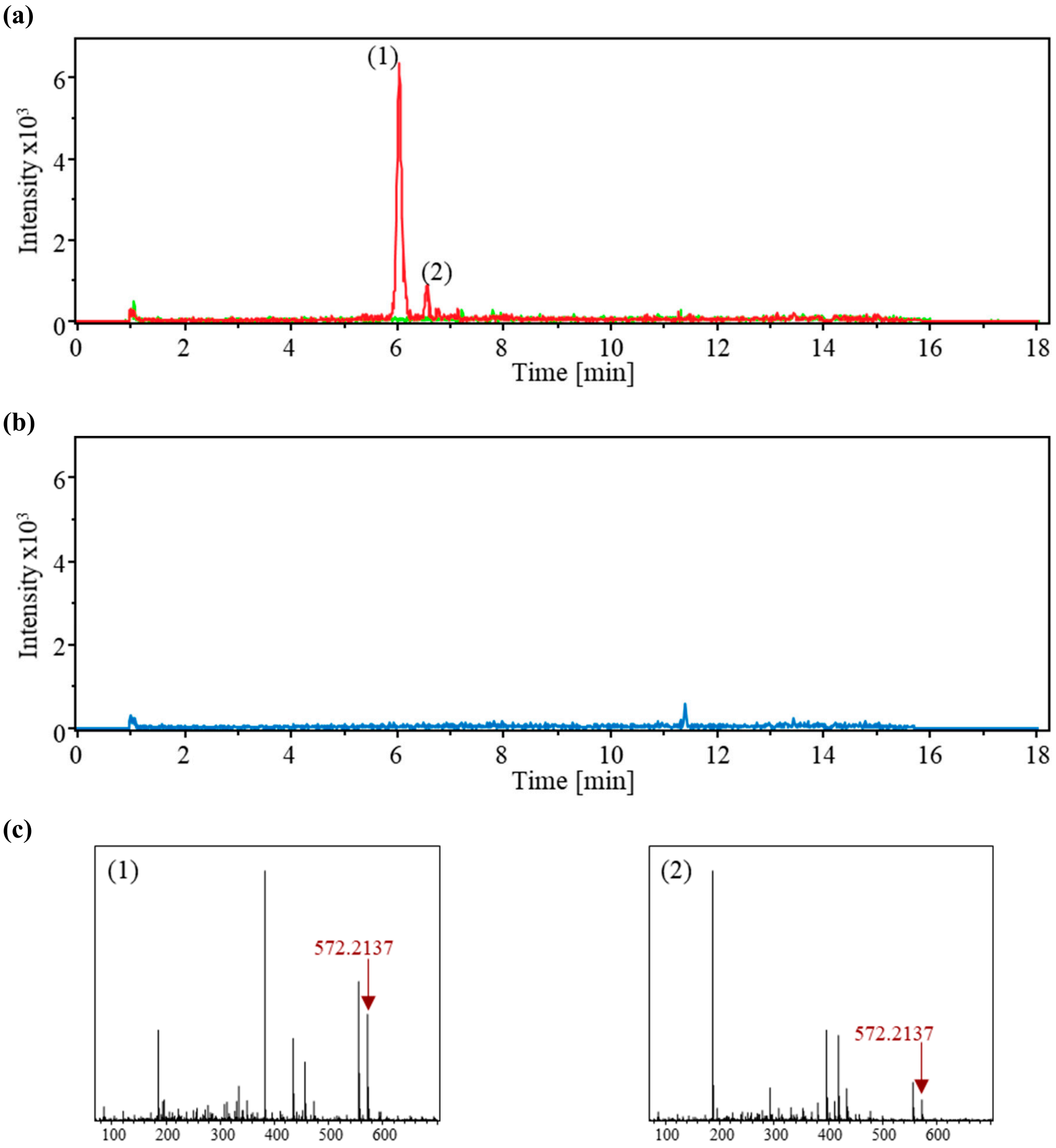

Figure S11. Identification of monohydroxylated and glucuronidated metabolites of **4p**. (a) Based peak chromatograms (m/z 572.2137) of monohydroxylated and glucuronidated metabolites of **4p** (Figure 8b 8) of samples incubated with mouse liver microsomal homogenates for 90 min at 37 °C in the presence of NADPH (green) and NADPH + UPGA (red) as cofactors for phase I and phase II metabolism enzymes. (b) Based peak chromatogram of **4p** treated analogously to (a) but without cofactors. (c) Mass spectra of monohydroxylated and glucuronidated metabolite species shown in (a). Metabolites were identified from these spectra via expected molecular formulas from Data Analysis Software (Bruker Daltonics) with errors below 10 ppm.
